# The Beyond-Human Natural World: Providing Meaning and Making Meaning

**DOI:** 10.3390/ijerph20126170

**Published:** 2023-06-19

**Authors:** Holli-Anne Passmore, Ashley N. Krause

**Affiliations:** 1 Department of Psychology, Concordia University of Edmonton, AW 236, Allan Wachowich Centre for Science, Research, and Innovation, 7128 Ada Boulevard, Edmonton, AB T5B 4E4, Canada; 2 Department of Psychology, University of Florida, 945 Center Drive, Gainesville, FL 32611, USA; ashleykrause@ufl.edu

**Keywords:** nature, nature connectedness, meaning in life, coherence, purpose, significance, place, committed actions for nature

## Abstract

Much academic and media attention has been focused on how nature contributes to psychological health, yet, most of this focus has been on happiness or hedonic well-being. Although numerous writers and researchers have linked connecting with nature as a pathway to meaning in life, an integrated overview has not yet (to our knowledge) been offered. Our manuscript is thus of both theoretical and practical importance with respect to finding meaning in life. In this hybrid commentary/review paper, we examine the link between meaning in life and relating to the beyond-human natural world. Through presenting supportive empirical research and interdisciplinary insights, we make the case that connecting with the natural world provides us with meaning in various ways. We discuss how nature is a common source of meaning in people’s lives and how connecting with nature helps to provide meaning by addressing our need to find coherence, significance/mattering, and purpose (the three aspects comprising the tripartite model of meaning life). We also consider how connecting with nature enhances our experiential appreciation for life, a fourth aspect of meaning in life recently proposed. Our discussion then expands to examining nature as a place of attachment. Going beyond how nature provides us with meaning, we consider how engaging in nature-based activities provides an avenue for many people to build meaningful lives. We close by considering how threats to nature are a threat to meaning in life.

## 1. Introduction

“*Those who come to the natural world for meaning will not go away unrewarded*”

—([1], p. 7).

Throughout history, humans have turned to nature when searching for meaning. Buddha attained enlightenment by sitting under a sacred fig tree; from the early centuries of the Common Era, individuals wandered in the mountains and by streams to contemplate the meaning of life. Frankl [2] wrote of experiences of natural beauty as one pathway to meaning in life. Philosophers such as Note [3] and Haybron [4] have written of nature experiences as inspiring meaningfulness in our lives. This persistent connection between the natural world and meaning in life begs the question, “How does nature provide us with a greater sense of meaning in life?”.

To explore this question, we first take a step back to situate meaning in life within the broader well-being literature. The hedonic branch of well-being is generally thought of as emotional well-being, that is, comprising happiness and other positive emotions [5,6]. The eudaimonic branch of well-being is generally thought of as comprising a range of markers such as authenticity, environmental mastery, personal growth, factors of self-determination (i.e., autonomy, competence, relatedness), social well-being, and purpose or meaning in life [6,7]. Within this framework, meaning in life sits squarely within a eudaimonic view of well-being [6,7,8,9]. (This does not mean that eudaimonic activities do not engender positive emotions; of course, they often do [9,10]). With specific respect to nature, a great deal of research has focused on how connecting with nature contributes to hedonic well-being, that is, happiness and other positive emotions (see reviews by [11,12]). Herein, we focus on links between relating to the beyond-human natural world and meaning in life. Before we begin our review, it is also important to clarify terms regarding (a) ‘time in/connecting with/exposure to/experiences in’ nature and (b) ‘connectedness to nature’ or ‘nature connectedness’. As Capaldi et al. [13] note, time in, connecting with, exposure to, and experiences in nature all refer to contact with nature—whether wild or urban, inside or outside, live or virtual. Such contact can be a one-time activity or experience, or it may involve repeated instances of contact of a longer duration. Nature connectedness or connectedness to nature, on the other hand, refers to a construct referring to one’s subjective sense of connection with the natural world. While highly correlated, contact with nature and the construct of nature connectedness are distinct aspects of the human–nature relationship.

## 2. Providing Meaning

**Figure 1:**
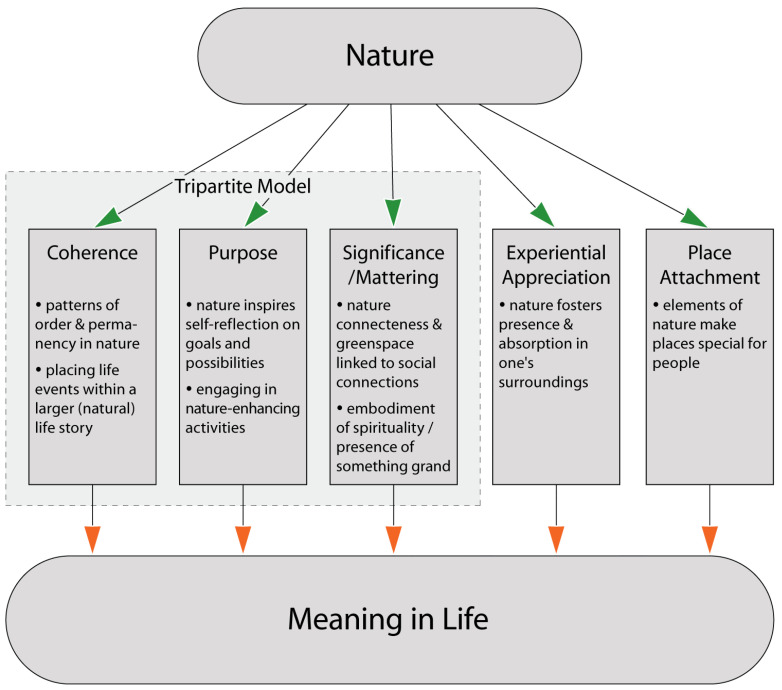
Nature pathways to meaning in life.

Overall, the essence of meaning is the connection [14,15] to something larger than oneself—be it other people, social movements, spirituality, or, as we discuss herein, to the beyond-human natural world. Below we delve into (and provide supportive research findings for) how relating to nature is linked to meaning in life in general and to each tripartite facet of meaning—coherence, significance/mattering, and purpose [16,17,18,19]—in addition to a more recently proposed fundamental facet of meaning, experiential appreciation [20] and examining nature as ‘place’. (See Figure 1 for a summary map).

### 2.1. Nature and Meaning in Life: In General

Empirically, nature has emerged as an important source of meaning in life in many research studies. In early research working towards developing inventories of sources of meaning in life, O’Connor and Chamberlain [21] found that a separate category pertaining to nature as a source of meaning in life was needed given that 53% of their participants noted relating to, or appreciating, nature. Reker [22] revised the Sources of Meaning Profile scale to include the category of “a relationship with nature” in order to improve the factor structure of the scale. Based on an accumulation of empirical findings, other researchers have also included nature (or nature-related activities such as gardening) as an important source of meaning in life in their inventories; these include the Sources of Meaning and Meaning in Life Questionnaire (SoMe; [23]) and the Schedule for Meaning in Life Evaluation (SMiLE; [24]).

Nature as a source of meaning in life has emerged in numerous qualitative studies. For example, in Reker and Woo’s [25] study of community-residing older adults, a relationship with nature emerged as a meaning source within the cluster they called a “self-transcendent meaning orientation”. Psychologists in Kernes and Kinnier’s [26] study rated the item “Nature and the environment bring meaning to my life” among the top ten items representing personal terrestrial meaning, that is, ways in which individuals create or discover meaning in their lives. Affinity with nature emerged as a source of meaning in life in Shoshtari et al.’s [27] study of meaning in life for a sample of Iranian university students. Nature emerged as a main category of sources of meaning in life in Steger and colleagues’ [28] study wherein participants submitted photos of sources of meaning in their lives. Sub-categories within this were mountains, flowers, seasons, and bodies of water. Nature was named as one of the top eight sources of meaning for people in the United Kingdom, Australia, France, New Zealand, and Sweden in Pew Research Centre’s 2021 [29] large-scale study of where people find meaning in life.

In line with qualitative findings, a growing body of quantitative research exists evidencing a link between relating to nature and enhanced meaning in life. This includes results from experimental studies. Hamann and Ivtzan [30] randomly assigned participants to the Rewild Your Life intervention program, which challenged participants to spend 30 min a day in nature for 30 days or to a wait-list control group. Participants in the 30-day Rewild Your Life program showed significant increases in meaning in life compared to the control group. Two-week nature interventions have also been found to boost meaning in life. In two 14-day studies, compared to the control groups, participants who had been assigned to spend more time in nature [31] or to simply notice how the everyday nature encountered in their daily routine made them feel [32] reported higher levels of meaning in life.

Even briefer exposure to nature has also been shown to enhance levels of meaning in life. In a series of field and laboratory studies involving spending a brief period of time in a park setting, watching nature videos, completing surveys in a room with indoor plants, and creating artwork using natural materials, Yang and colleagues [33] found that participants randomly assigned to the nature conditions (compared to the control conditions) reported higher levels of meaning in life.

Additionally, in correlational research, nature connectedness (one’s subjective sense of connection with the natural world [19]) has emerged as a significant correlate of meaning or purpose in life in several studies [34,35,36,37,38,39] (see also scoping review by [40]). Richardson et al. [41] reported that nature connectedness uniquely accounted for 25% of explained variance in feeling that life is worthwhile (a proxy indicator of meaning in life) when considering nature connectedness, engaging in simple nature activities, and time in nature.

### 2.2. Nature Pathways to Meaning in Life: Coherence

With respect to meaning in life, coherence refers to an intuitive feeling or cognitive understanding that the world makes sense [42,43]. This includes identifying with elements of stable patterns and permanency [44], feeling that one’s life makes sense [18] and fits within a larger scheme [45], and feeling that “things are as they ought to be” ([16] p. 206). The coherence aspect of meaning is, however, generally considered as primarily a cognitive component in personal meaning [46,47].

Even in this time of climate change, patterns of order and permanency exist in nature: the sun still rises each day in the east; shoots still push through the soil in the spring; summer still follows spring, followed by autumn, and then winter. Indeed, non-randomness—patterns and regularities—and unity are inherently part of the physical forces which shape the beyond-human natural world and are evident throughout nature, from snowflakes to sand ripples, clouds to mountain cliffs [48]. Observing and reflecting upon such natural patterns have been noted to provide comfort and a sense that the world makes sense [49,50,51] (see also [52]). As Weil [53] wrote, “The natural world is built upon common motifs and patterns. Recognizing patterns in nature creates a map for locating yourself in change, and anticipation what is yet to come”.

Although sparse in number, empirical studies lend support to the supposition that nature can act as a pathway to the coherence aspect of meaning in life. Qualitative reports from clinical approaches indicate that integrating nature into therapy via exposure to nature or the use of nature metaphors helps clients to make sense of and find meaning in life events by placing life events within a larger life story [54,55]. Lipowski et al. [56] reported that nature connectedness correlated with dimensions of coherence comprised of comprehensibility, manageability, and meaningfulness.

### 2.3. Nature Pathways to Meaning in Life: Significance/Mattering

Significance or mattering refers to a sense that one’s life has inherent value [18], that one matters (and belongs) at a social (human) level [57] and/or on a cosmic or grand level [17]. (Significance and matter in the context of meaning also refer to a sense that one’s actions make a difference in the world [58]. We discuss this in Section 3 on “Making Meaning”). This aspect of meaning has more of an emotional flavour [46,47].

#### 2.3.1. Social Significance/Mattering

Petersen and colleagues [59] argued that conceptual similarities exist between, and common emotions underpin, social connectedness and nature connectedness. Supporting this stance are empirical findings of a positive correlation between nature connectedness and social well-being or social cohesion [36,60,61] (see also meta-analysis by [62]). A link between the amount of green space in neighbourhoods and the strength of social ties among neighbours has also been evidenced [63,64,65]. Building on such data, and bolstered by a comprehensive review of a wealth of supportive literature, Leavell et al. [66] proposed nature-based social prescribing as a way to boost social connections and meaning.

Furthermore, grand nature prototypically evokes awe [67], a self-transcendent emotion that pulls people into the social collective via the salience of one’s large-group identity [68,69,70]. Results from numerous studies have demonstrated that exposure to awe-evoking nature boosts social connections (see [71] for a review). These boosts to social connection presumably enhance a sense of social significance/mattering aspect of meaning in life.

#### 2.3.2. Cosmic Significance/Mattering and Spirituality

In addition to social mattering, as noted above, the significance/mattering aspect of meaning in life also comprises cosmic mattering, a sense of belonging to something vast, beyond humanity. Here, too, supportive evidence has emerged for nature as a pathway to this aspect of meaning in life. In their study examining sources of meaning in life, O’Connor and Chamberlain [21] noted how participants frequently spoke of nature as “providing a connection with cosmic meaning” (p. 467). An abundance of studies have since demonstrated that viewing and experiencing awe-inspiring nature evokes feelings of being in the presence of and connected to something greater than oneself [72]. Everyday nature has also been shown to evoke this sense of “transcendent connectedness”(feeling connected to other humans, to nature, and to life in general). In a series of three studies, participants who merely noticed how the everyday nature they encountered in their daily routines made them feel reported significantly higher levels of transcendent connectedness compared to those in control conditions [32,73,74].

William James [75,76] noted how experiences in nature can bridge a sense of connectedness between oneself and the entire universe, a sense of unity with all things he referred to as a spiritual identity. Bethelmy and Corraliza [77] proposed that spiritual responses elicited by nature are a specific type of transcendent emotion. These grand, transpersonal feelings of communion can be viewed as an aspect of nonreligious spirituality [78], a particularly potent source of mattering [79].

Results from several studies demonstrate a significant correlation between nature connectedness and spirituality [80,81,82] (see also scoping review by [40]). Exposure to nature, particularly wild nature, often triggers intense spiritual experiences [77,83,84,85,86,87]. Qualitative studies and the literature within religious studies and other disciplines suggest that nature is often viewed as an embodiment of spirituality [85,88,89,90], such that nature is commonly included in measures of spirituality (e.g., [91,92,93]) or sources of spirituality [94].

### 2.4. Nature Pathways to Meaning in Life: Purpose

Within a meaning framework, purpose refers to having core goals and aims in life [18]. Understanding one’s purpose in life necessarily requires time for self-reflection [18,95,96,97]. Various writers and researchers have posited that experiences in nature may provide space for reflection and perspective-making [88,98]. For example, Kaplan and Kaplan [99] asserted that experiences in nature tend to elicit “reflection on one’s life, on one’s priorities and possibilities, on one’s actions and one’s goals” (p. 197).

Results from studies support these suppositions. In studies examining places to engage in self-reflection, natural spaces have been notably preferred [100,101,102,103]. Regaining a sense of self and the ability to self-reflect have been reported as benefits by participants in farm- or green-care-based therapy programs (beyond the benefits of engaging in meaningful work and social belongingness cultivated by participating in the program) [104]. Across three experimental studies, Mayer et al. [105] found that participants who were exposed to nature (vs. control conditions) reported greater ability to self-reflect.

Broadly speaking, purpose is about connecting one’s actions to a meaningful and purposeful whole [20]. In this vein, directly linking nature and purpose are findings from numerous studies demonstrating that engaging in nature-enhancing and nature-protective activities provides purpose to people’s lives. We discuss these findings in the Section 3 on “Making Meaning”.

### 2.5. Nature Pathways to Meaning in Life: Experiential Appreciation

Building on the tripartite model of meaning as comprising coherence, significance/mattering, and purpose [16,17,18,19], Kim and colleagues [20] have proposed a fourth fundamental aspect of meaning in life: experiential appreciation. That is, appreciating the beauty of life by valuing and being present in the moments of one’s life experiences. One way in which they tested this theory (Studies 5 and 6) was by randomly assigning participants to watch an awe-inspiring nature video (vs. control videos). Results indicated that watching awe-inspiring nature boosted meaning in life via an indirect effect of enhanced experiential appreciation. Although further investigation is needed in this area, these initial findings parallel previous findings by Debats [106], wherein appreciation of life itself, described as “plants, trees, birds … the sounds of the birds, the sea, a brooklet”, emerged as a top source of meaning in life (p. 39), and findings by Wright and Matthews [107] who discussed how meaningful nature experiences led to increased awareness and sensory perception, along with intense emotional experiences.

Several theories provide support for nature fostering experiential appreciation, as noted by Ballew and Omoto [108] in reporting their findings of how nature fosters absorption in one’s surroundings (see also [109]). Grounding much of this is the Biophilia Hypothesis, which states that, as humans, we have an evolved proclivity to affiliate with the greater-than-human natural world and to respond to nature with emotional intensity [110,111]. Burns [55] detailed how certain characteristics of stimuli found in natural environments—variety, intensity, motion—provide for a sense of enjoyable appreciation. Building on this, Perceptual Fluency Account [112] and Attention Restoration Theory [99] speak to how the fractal geometry of nature supports a state of effortless fascination and absorption, ultimately leading to an appreciation of our experiences in nature.

### 2.6. Nature Pathways to Meaning in Life: A Place of Attachment

The idea of nature as a place has surfaced in various writings. Firth [113] argued that the relationship with place is meaningful at an individual and collective level and that historically ‘place’ includes its flora and fauna. Richardson [114] wrote of how nature is a portable place of interconnection, in that nature can be found in many places, including urban environments or “metro nature” [115]. Echoing this, Bush and colleagues [116] stated that “nature is always present in place” (p. 40).

As noted above, the Biophilia Hypothesis puts forth that, as humans, we have an inherent proclivity to affiliate with nature [110,111]. While over a century prior, Alexander von Humboldt (1769–1859) wrote of how “Nature everywhere speaks to man in a voice … that is familiar to his soul” (as cited in [117], ebook p. 89). It is perhaps this sense of inherent familiarity that makes nature such a powerful place of attachment and meaning. In their report on why places matter to people, the National Trust in the United Kingdom [118] noted that it was often elements of nature that connected people to a place and made it special for them. Wilderness values, with respect to the importance of landscape valuing, have emerged elsewhere as particularly important predictors of place attachment [119]. Nature bonding, in fact, was included as a distinct dimension of place attachment by Raymond and colleagues [120].

Feeling connected to a place has been found to relate to feeling that one’s life is worthwhile [118]; results which are substantiated by experimental results demonstrating that place attachment generates meaning in life [121,122]. Further, Basu and colleagues [123] found that place attachment mediated the relationship between nature connectedness and well-being, measured in part by items referring to purpose and meaning in life. Two meta-analyses have linked nature connectedness with pro-environmental behaviour [124,125] (see also [126]), and attachment to natural places has been found to predict pro-environmental behaviour [127,128,129]. Thus, nature connectedness and nature as a place of attachment come together in making meaning in people’s lives, a topic to which we now turn.

## 3. Making Meaning

Beneficence—a sense of having a prosocial impact—has been proposed not only as a basic human psychological need, but also as an important pathway to meaning in life [130]. As noted above, significance and mattering in the context of meaning also encompass a sense that one’s actions make a difference in the world [58]. Contributing to the larger world—committing to pro-social actions, goals, and objectives beyond one’s personal needs—is, in fact, central to meaning in life [15,130,131]. One class of benevolent pro-social actions that provide purpose, significance, a sense of coherence, and overall meaning to individual’s lives are committed actions for nature/pro-environmental activities (e.g., gardening, recycling, use of environmentally friendly products, energy-saving practices, and nature-based volunteering).

Meta-analytic findings provide evidence of a robust link between pro-environmental behaviour and meaning in life [132] (see also [133,134]), suggesting that meaning in life is an important driver for committing to actions for nature. Qualitative findings from studies examining motivations of committed actors for nature have clearly and consistently shown that such goals and actions are undertaken because they provide meaning to people’s lives [135,136,137]. In their study of committed community gardeners involved in the collaborative growing of produce, Quested and colleagues noted how a strong sense of meaning was evident and how these gardeners found satisfaction in knowing that they were making a difference in others’ lives (thus reflecting the significance/mattering aspect of meaning in life). Molinario and colleagues reported that committed actors for nature “defined their activity and commitment as motivated by the need to do something useful, which makes their life meaningful, and lends them significance” (p. 1147). Motives reflecting significance/mattering, coherence, and purpose were evident in van den Born and colleagues’ findings, with committed actors frequently expressing “a life-directing desire to make a difference in the world” (p. 849; reflecting the purpose and significance/mattering aspects of meaning in life) and “an active awareness of the need for actions to fit into the greater life-story” (p. 849; reflecting the coherence aspect of meaning in life). Moreover, van den Born et al. concluded that these individuals engage in pro-environmental behaviour not only because it is a source of meaning in their lives but also because they feel a strong sense of nature connectedness.

## 4. Threats to Nature—Threats to Meaning

In the tripartite model of meaning in life, coherence includes feeling that “things are as they ought to be” ([16], p. 206). But, things are most definitely *not* how they ought to be with respect to the natural world: one million plants and animals are threatened with extinction at a rate that is accelerating tens to hundreds of times higher than the average over the last 10 million years [138]. Deep patterns in nature are now noticeably disrupted due to anthropogenic climate change [139], with widespread abrupt ecological disruption predicted to occur between 2030 and 2100 in the world’s most biodiverse ecosystems [140].

These threats to nature are also a threat to meaning. Molinario et al. [135] suggested that people who live in places with severely degraded natural environments are likely to experience a loss of significance. Experiencing dramatic negative environmental transformations in places they were attached to preceded a search for meaning and significance in 44% of the committed actors for nature in Molinario and colleagues’ study. Budziszewska and Jonsson [141] wrote of how the current climate crises bring to the surface questions of meaning in life for many people. Morgan and colleagues [142] noted how feelings of meaninglessness emerge when people experience strong feelings of eco-anxiety (i.e., persistent concern over biodiversity loss, climate change, and degradation of the natural environment), and Pihkala [143] framed linking eco-anxiety research with research on meaning as crucial.

Steger [144] referred to meaning in life as “the web of connections, understandings, and interpretations that help us comprehend our experience” (p. 165). As Passmore et al. [145] noted, when the threads of our connections become “frayed or broken, as is occurring with climate and ecological disruptions, our lives seem less connected, less coherent, less significant—less meaningful” (p. 143). For many individuals experiencing these threats to meaning elicited by the devastating threats to nature we are bearing witness to, becoming involved in climate action becomes a source of meaning in life [135,141]. Taking action for our planet by engaging in pro-conservation and pro-environmental behaviours, becoming committed actors for nature, is recommended as a meaning-focused (and constructive) strategy for coping [146], as is fostering one’s connection to nature [145].

## 5. Future Research Directions

Future research is warranted to build on the above-reviewed findings. For example, while studies have consistently found that nature connectedness is significantly and positively correlated with meaning in life as a whole, expanded research is needed to assess the relationship between nature connectedness and each aspect of meaning in life. New measures of meaning could be utilized which have been developed to capture these aspects, such as the Multidimensional Existential Meaning Scale [17], the Three-Dimensional Meaning in Life Scale [19], and the Perceived Mattering Questionnaires (Perceived Overall Mattering; Perceived Interpersonal Mattering; Perceived Cosmic Mattering; [147]), and Experiential Appreciation [20]. Different measures of nature connectedness could also be used in the study to capture nuanced aspects of the nature connectedness construct and to guard against findings being merely an artifact of one specific measure. Given the link between nature connectedness and meaning in life, randomized control experimental studies are suggested wherein nature connectedness is manipulated (i.e., intervention studies specifically aimed at boosting nature connectedness), and meaning in life is subsequently assessed as a direct result. Lumber and colleagues’ [148] proposed five pathways to nature connectedness could be used as a base for such research.

Building on studies that demonstrated that brief exposure to nature videos boosted meaning in life (e.g., [33]), it would be interesting to see if, conversely, exposing participants to videos of degraded nature reduces meaning in life, in particular the coherence and experiential appreciation aspects. Along this line of investigation, it would be interesting to examine the relationship between eco-anxiety and meaning in life. Limited research exists in this area at this point, and what research does exist has provided inconsistent findings [149,150]. Moderating variables could be explored to assess if the relationship between eco-anxiety and meaning in life (in general and its components) depends upon levels of nature connectedness and/or degree of engagement in pro-nature activities. Another branch of research that could be explored would be to study if eco-anxiety acts as a call to meaningful engagement with nature and meaningful collective action (as proposed by [151]).

A number of studies are needed to more thoroughly assess how engagement with nature can boost meaning in life via the enhancement of experiential appreciation. Expanded replication studies could be conducted based on Ballew and Omoto’s [108] and Sato and Connor’s [109] studies on how nature fosters absorption, with the addition of measuring meaning in life (in general and its components). Perhaps some ways of engaging with nature yield differential effects on meaning in life or on different aspects of meaning in life. It is possible that meaningful nature experiences may act as a catalyst sparking a more generalized experiential appreciation in other areas of life.

Although much literature and several qualitative studies have clearly evidenced that people turn to nature when needing to reflect and when searching for meaning, there is a dearth of quantitative studies in this regard. Research utilizing the Search for Meaning subscale of the Meaning in Life Questionnaire [152] would be helpful to quantify if searching for meaning decreases when people affiliate with nature.

With respect to place attachment, nature, and meaning in life, there are a number of directions future research could proceed. One such direction could be to build on work by Krause and colleagues [121,122], which evidenced that people in places of attachment show increased meaning in life, to test if this effect is enhanced in built places which incorporate elements of biophilic design.

Lastly, it bears worth mentioning that results from large population studies provide evidence of the physical health benefits that living near greenspace affords [153,154,155]. Most recently, in their meta-analysis and systematic review (participant N = 8,324,652 across seven countries), Rojas-Rueda and colleagues [156] reported a significant inverse relationship between living in greener urban areas and all-cause mortality (controlling for socioeconomic status). They recommended that increasing and managing nature spaces be considered a strategic public health intervention (see also [154,157]) and that nature be considered as part of public health policy. Large population studies also evidence the physical health benefits that people with high (vs. low) levels of meaning in life enjoy (see reviews [158,159]). Research is needed to inform public health policies which capitalize on combining these findings for synergistic impact.

## 6. Conclusions

In the old reality of biblical times, people depended on the Earth, on the nature they found around them, for both life *and* meaning [50]. Today, despite our mainly urban lifestyles centered around technology, we still rely on nature for life, *and* we still find meaning in the natural world. As presented in this article, we turn to nature as a source of meaning in our lives, to find comfort and coherence in nature’s patterns and elements of permanency. We seek nature as a place to foster meaningful connections with other people and with the sacred, to reflect on our goals and purposes in life, and to find perspective. We find joy in appreciating the beauty of nature in its myriad forms and how it awakens our senses to the greater experiential appreciation of life. We turn to nature for meaning.

At its essence, meaning is about connection, about relationships. Cooper [160] defined meaning as relating to something larger than or outside oneself. James [161] expanded on this by using the example of explaining the meaning of a particular sequence of musical notes by how “it fits into the movement of the symphony to which it belongs” (p. 612). Perhaps ultimately, our search for meaning is a search for how we fit into the larger symphony of life, into the natural world of which we are a part, regardless of our varying tendencies to view ourselves as separate from nature. As Firth [113] speculated: “Meaningful lives that have no connection with the natural world are very hard to imagine” (p. 149).
